# Positive selection on hemagglutinin and neuraminidase genes of H1N1 influenza viruses

**DOI:** 10.1186/1743-422X-8-183

**Published:** 2011-04-21

**Authors:** Wenfu Li, Weifeng Shi, Huijie Qiao, Simon YW Ho, Arong Luo, Yanzhou Zhang, Chaodong Zhu

**Affiliations:** 1 Key Laboratory of Zoological Systematics and Evolution, Institute of Zoology, Chinese Academy of Sciences, Beijing 100101, China; 2 Graduate University of Chinese Academy of Sciences, Beijing 100049, China; 3 UCD Conway Institute of Biomolecular and Biomedical Sciences, University College Dublin, Dublin 4, Ireland; 4 Key Laboratory of Animal Ecology and Conservation Biology, Institute of Zoology, Chinese Academy of Sciences, Beijing 100101, China; 5 School of Biological Sciences, University of Sydney, Sydney NSW 2006, Australia

## Abstract

**Background:**

Since its emergence in March 2009, the pandemic 2009 H1N1 influenza A virus has posed a serious threat to public health. To trace the evolutionary path of these new pathogens, we performed a selection-pressure analysis of a large number of hemagglutinin (HA) and neuraminidase (NA) gene sequences of H1N1 influenza viruses from different hosts.

**Results:**

Phylogenetic analysis revealed that both HA and NA genes have evolved into five distinct clusters, with further analyses indicating that the pandemic 2009 strains have experienced the strongest positive selection. We also found evidence of strong selection acting on the seasonal human H1N1 isolates. However, swine viruses from North America and Eurasia were under weak positive selection, while there was no significant evidence of positive selection acting on the avian isolates. A site-by-site analysis revealed that the positively selected sites were located in both of the cleaved products of HA (HA1 and HA2), as well as NA. In addition, the pandemic 2009 strains were subject to differential selection pressures compared to seasonal human, North American swine and Eurasian swine H1N1 viruses.

**Conclusions:**

Most of these positively and/or differentially selected sites were situated in the B-cell and/or T-cell antigenic regions, suggesting that selection at these sites might be responsible for the antigenic variation of the viruses. Moreover, some sites were also associated with glycosylation and receptor-binding ability. Thus, selection at these positions might have helped the pandemic 2009 H1N1 viruses to adapt to the new hosts after they were introduced from pigs to humans. Positive selection on position 274 of NA protein, associated with drug resistance, might account for the prevalence of drug-resistant variants of seasonal human H1N1 influenza viruses, but there was no evidence that positive selection was responsible for the spread of the drug resistance of the pandemic H1N1 strains.

## Background

As of August 1^st^, 2010, the pandemic influenza H1N1 2009 had caused at least 18,449 deaths worldwide in more than 214 countries [1]. It has been reported that influenza A viruses are capable of infecting 30% of the world population within a single month owing to their rapid inter-personal transmission ability, thus posing a serious threat to public health [2]. Therefore, there are compelling reasons to investigate the molecular evolution of H1N1 influenza A virus to improve its prevention and control.

Influenza A virus belongs to the Orthomyxoviridae family, with a negative-sense single-stranded RNA genome composed of eight gene segments [3]. Hemagglutinin (HA) and neuraminidase (NA) are the two envelope glycoproteins that are responsible for attaching the virions to the host receptors, determining pathogenicity, and releasing newly produced viral particles. To date, influenza A virus has been classified into 16 HA and 9 NA subtypes and more than 100 HA-NA combinations have been identified in avian hosts [4]. Notably, HA is cleaved into HA1 and HA2, with HA1 being the major target of human immunity against influenza A virus [5,6]. Meanwhile, mutations at NA sites are associated with drug resistance; for example, H274Y and N294S confer resistance to oseltamivir [7].

The comparison of synonymous and nonsynonymous substitution rates is the most common approach used to determine the existence of positive selection. Interpretations are normally made with reference to the nonsynonymous/synonymous substitution rate ratio (ω = *d*_*N*_*/d*_*S*_) [8], where the rates *d*_*N *_and *d*_*S *_are the numbers of nonsynonymous and synonymous substitutions per site, respectively. The ratio ω measures the selective pressure at the protein level. Values greater than 1 suggest that nonsynonymous mutations offer fitness advantages to the protein (individual) and have higher fixation probabilities than synonymous mutations [9].

There have been several studies investigating positive selection on H1N1 influenza viruses. Wolf et al. [10] reported that from 1995 to 2005 there was no clear selection pressure acting on seasonal human H1N1 HAs. However, Shen et al. [11] analysed H1N1 influenza viruses isolated from 1918 to 2008 and found strong diversifying (positive) selection at HA1 156 and 190. The residues 190 and 225 are critical determinants of the receptor-binding specificity of A/H1N1 HA, with human viruses favouring D190/D225, swine viruses favouring D190/G225 and avian viruses favouring E190/G225 (D190 means that the amino acid at position 190 is D, aspartic acid. This notation is used throughout this paper.) [12]. Recently, Furuse et al. [13] reported that selection pressures acted differently on the pandemic 2009, seasonal human and swine H1N1 strains. In addition, it has been reported that positive selection was responsible for the spread of the oseltamivir-resistant variants of both seasonal H1N1 and pandemic 2009 H1N1 influenza viruses [14].

Although the above studies are helpful in explaining the evolutionary characteristics of H1N1 influenza viruses, some questions remain. First, although there have been many reports concerning the positive selection pressures on the HA and NA proteins of human H1N1 influenza, the relationship between the positively selected sites and antigenic variation of the virus remains unclear [11,14]. Second, the mature HA protein has two subunits, HA1 and HA2, connected by disulfide linkage [5]. Some previous authors have also studied the HA2 subunit [10,13]. For example, Wolf et al. [10] performed a positive-selection analysis of the full-length HA gene sequences of the H3N2 and H1N1 to study the interpandemic evolutionary trend of human influenza A. However, there has been a lack of detailed description of a site-by-site positive-selection analysis of this subunit. Third, swine H1N1 influenza viruses have evolved into two separate lineages, the North American lineage and the Eurasian lineage [15,16]. These two lineages were the respective sources of the HA and NA of the pandemic 2009 virus [17]. However, Furuse et al. [13] did not distinguish between them. Thus, positive-selection pressures on the two swine lineages are not clear. Fourth, H1N1 influenza viruses also circulate in birds. However, no analysis of positive selection has been conducted for avian H1N1 influenza viruses.

To address these questions, we performed a positive-selection analysis of full-length HA and NA genes of H1N1 influenza viruses available in GenBank. Our analysis offers some insight into the evolutionary trends of H1N1 influenza viruses.

## Results

### Phylogenetic analysis

The HA phylogenetic tree constructed using Dataset1 contained five clusters of lineages (Additional file 1, Table 1). Cluster 1.1 included strains isolated from avian hosts. Cluster 1.2 mostly consisted of strains from North American swine. Cluster 1.3 largely contained strains from Eurasian swine, whereas cluster 1.4 was the seasonal human H1N1 lineage. Cluster 1.5 mainly included viruses isolated from the pandemic 2009 strains. The pandemic 2009 strains were more closely related to those from North American swine.

**Table 1:** Positively selected sites in hemagglutinin from viruses from different clusters

Dataset	Description	Number of sequences	Length of alignment (bp)	Number of sites under positive selection		**Positively selected sites**^**1**^			
						
						HA1		HA2	
				
				SLAC	FEL	SLAC	FEL	SLAC	FEL
1.1	Avian strains	75	1647	0	0				
1.2	North American swine strains	196	1647	0	1		138		
1.3	Eurasian swine strains	90	1647	0	1				399
1.4	Seasonal human strains	1404	1647	8	8	82^B^,94^B^, 141^B^,162^BG^,186^B^,187^BR^,222^BR^	82^B^,94^B^,160^BG^,162^BG^,186^B^,187^BR^,222^BR^	451^T^	451^T^
1.5	Pandemic 2009 human strains	1891	1647	7	9	186^B^,197,203,205^B^,222^BR^,223^B^,261^BT^	186^B^,197,203,222^BR^,261^BT^		411^T^,451^T^,460^T^,530^T^

^1^In these columns, B indicates that the site lies in the B-cell antigenic regions [18]. G means that it is a potential glycosylation site. T indicates that the site lies in the T-cell antigenic regions [19]. R indicates that it is a receptor-binding site [11]. We use the same numbering strategy as Deem and Pan [18] and start numbering from the amino acids DTLC.

The phylogenetic tree of NA genes revealed relationships similar to those observed in the HA tree, with one exception (Additional file 2). The pandemic 2009 strains were related to viruses from the Eurasian swine lineage rather than the North American swine lineage.

### Analysis of positive selection

**Figure 1:**
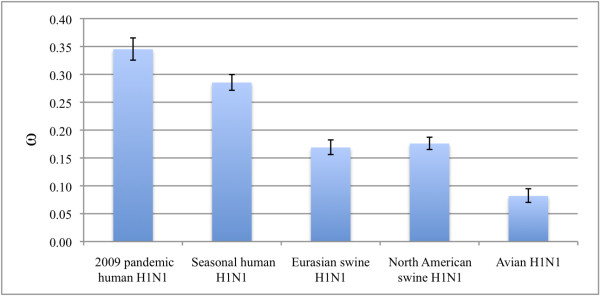
**Selection pressure on HA genes from different clusters**. Selection pressures for the global ω of HA genes of each cluster. Error bar shows 95% confidence interval.

**Figure 2:**
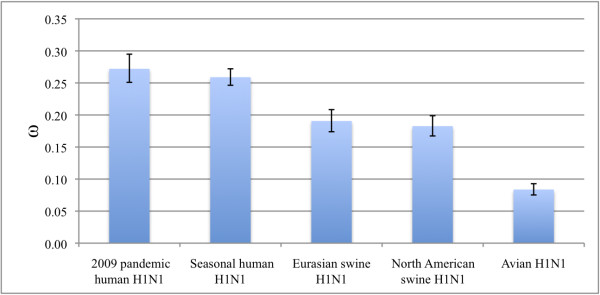
**Selection pressure on NA genes from different clusters**. Selection pressures for the global ω of NA genes of each cluster. Error bar shows 95% confidence interval.

Global ω values showed similar results for both HA and NA. The global ω values were below 1.0 for all five clusters, which indicates that there is no detectable positive selection on the gene as a whole (Figures 1 and 2). The ω values for human strains were higher than those for viruses from other hosts. In particular, the ω values of the pandemic 2009 viruses were the highest. ω values for the seasonal human H1N1 and the pandemic 2009 H1N1 lineages were higher than those for viruses from Eurasian and North American swine which, in turn, were similar to each other. Avian strains yielded the lowest ω value.

Further site-by-site tests of positive selection helped to identify the specific sites that were not detected by the global positive-selection analysis. Results obtained by the single likelihood ancestor counting (SLAC) and fixed-effects likelihood (FEL) methods were very similar (Table 1). Specifically, for HA genes, positive selection has been detected to act on viruses belonging to different clusters, North American swine, Eurasian swine, seasonal human, and the pandemic 2009, with each having 1, 1, 8, and 9 positively selected sites, respectively, in the FEL analysis (Table 1). However, there was no evidence of any positively selected sites in the avian cluster. Among the positively selected sites in viruses from the seasonal human cluster, 7 positions are located in HA1 and all of them fall within B-cell antigenic regions, while 1 position is located in the T-cell antigenic region in HA2 [18,19]. In particular, positions 160 and 162 are potential glycosylation sites and positions 187 and 222 are associated with receptor-binding ability [11]. Furthermore, for the pandemic 2009 isolates, 5 sites are located in HA1 and 4 in HA2. Among them, positions 186, 222 and 261 lie in the B-cell antigenic regions, while 261, 411, 451, 460 and 530 lie in the T-cell antigenic regions [18,19]. Furthermore, positions 160, 186, 187, 222 in HA1, and 399 in HA2 are related to the host shift of the viruses from birds to humans [20]. Overall, for the seasonal human lineage (1.4), the FEL analysis shows that all 8 of the positively selected sites lie within the T-cell and/or B-cell antigenic regions, whereas for the pandemic H1N1 lineage (1.5), 7 of the 9 sites under positive selection are located within the T-cell and/or B-cell antigenic regions.

The SLAC analysis of the NA gene sequences showed fewer sites under positive selection than the FEL analysis (Table 2). However, many of the positively selected sites detected by the SLAC method were also found to be under positive selection in the FEL analysis. In the FEL analysis, 7, 1, 6, and 2 sites were found to be positively selected in NAs of viruses from North American swine, Eurasian swine, seasonal human, and the pandemic 2009 clusters, respectively (Table 2). No site was detected to be under positive selection for cluster 2.1, which was mainly composed of isolates from birds. Notably, positions 46, 53 and 453 were positively selected for viruses from North American swine. These positions are located in the T-cell antigenic regions, while position 339 lie in the B-cell antigenic regions. Positions 46 and 339 were associated with host adaptation after the virus was introduced from birds to humans and position 46 is also a potential glycosylation site [20]. Position 46 is also a potential glycosylation site. Among the positively selected sites for strains from the seasonal human cluster, positions 344 and 365 are situated in both B-cell antigenic regions, and position 365 is also a glycosylation site [21]. Overall, the FEL analysis shows that 2 of the 6 positively selected sites lie in the B-cell antigenic regions for the seasonal human lineage and 1 of the 2 positively selected lies in the T-cell antigenic region for the pandemic H1N1 lineage. Positions 365 and 382 have been reported to be involved with the host shift of the virus [20]. Two positions, 35 and 453, were positively selected for NAs of the pandemic 2009 strains. Position 453 lies in the T-cell antigenic regions [19]. It should be noted that position 274 (numbering 275 in this study), which confers drug resistance [7], was positively selected for seasonal human H1N1 virus. At this position, 1336 sequences (accounting for ~77% of all seasonal human H1N1 viruses) possessed histidine, while 398 sequences had tyrosine. However, there was no evidence of positive selection acting on this position of the pandemic H1N1 viruses, in which 1372 (~98%) sequences possessed histidine and only 24 sequences (less than 2%) had tyrosine.

**Table 2:** Positively selected sites in neuraminidase from viruses from different clusters

Dataset	Description	Number of sequences	Length of alignment (bp)	Number of sites under positive selection		**Positively selected sites**^**1**^	
				
				SLAC	FEL	SLAC	FEL
2.1	Avian strains	137	1368	0	0		
2.2	North American swine strains	171	1368	3	7	46^TG^, 396^B^,453^T^	46^TG^,53^T^,75,127,285,339^B^,453^T^
2.3	Eurasian swine strains	92	1368	0	1		75
2.4	Seasonal human strains	1735	1371	5	6	84,151,222,275^A^,382	84,151,275^A^,344^B^,365^BG^,382
2.5	Pandemic 2009 human strains	1397	1368	0	2		35,453^T^

^1^In this column, B indicates that the site lies in the B-cell antigenic regions [21]. G means that it is a potential glycosylation site. T indicates that the site lies in the T-cell antigenic regions [19]. We start numbering from the amino acids MNPN. A means that the site is associated with drug-resistance [7].

### Analysis of differential selection

Differential selection was found to act on 16, 8 and 6 sites on HA1, HA2 and NA, respectively, between seasonal human H1N1 and the pandemic 2009 human strains (Table 3). These differentially selected sites might have significant biological functions. For HA1, HA2 and NA, 10 out of 16, 4 out of 8 and 1 out 6 differentially selected sites lie in T-cell and/or B-cell antigenic regions. For example, positions 34, 86, 94, 153, 160, 187, 202, 224, 237 and 302 in HA1 are located within B-cell antigenic regions. Moreover, positions 34, 153, 250 in HA1, 430, 473, 527, 541 in HA2, and 52 in NA are located within T-cell antigenic regions. In particular, differential selection has resulted in distinct amino acid polymorphism at some positions, such as positions 86, 94, 153, 160, 202, 203, 234, 250, 302, 374, 399, 473 and 527 in HA, and positions 52 and 257 in NA (Table 3). For example, at position 160 in HA, almost all the pandemic strains had K, with only a single exception, whereas more than 95% (n = 1345) of the seasonal H1N1 strains had N.

**Table 3:** Sites under differential selection between isolates from seasonal human and the pandemic 2009 clusters

Protein	**Position**^**1**^	P-value	**Amino acid polymorphism**^**2**^	
			
			Pandemic 2009 strains	Seasonal human strains
HA	34^BT^	0.00286	E1886/G2/X2/K1	E1404
	39	0.00054	G1887/E3/R1	G1404
	86^B^	0.00233	D1856/G31/N2/Y1/E1	E1383/K19/G1/Q1
	94^B^	0.00014	D1885/N4/E1/X1	Y769/H574/D38/N19/Q2/A1/R1
	153^BT^	0.00786	K1891	G1132/E202/R28/K20/X18/V4
	160^BG^	0.00073	K1890/E1	N1345/K27/X12/S7/T7/D2/E2/A1/I1
	187^BR^	0.00445	D1884/G5/X2	D1072/X135/N132/V30/E23/G9/A2/I1
	197	0.00073	A1851/T23/S17	A1401/T3
	202^B^	0.00318	G1886/X4/W1	V1378/L17/A5/M4
	203	0.00828	T1341/S542/X7/A1	S1392/T10/F1/X1
	224^B^	0.00973	E1885/Q1/X5	E1342/A45/H5/P5/K4/T2/S1
	234	0.00628	V1883/I7/L1	L1404
	237^B^	0.00445	G1890/X1	G1379/R19/E6
	250^T^	0.00002	V1884/I4/A2/L1	A1404
	282	0.00653	P1872/L10/S7/T1/X1	P1404
	302^B^	0.00098	K1880/E8/N1/T1/R1	E1403/G1
	339	0.00174	G1888/R2/E1	G1404
	374	0.00550	E1608/K275/G8	G1398/R4
	391	0.00564	T1888/A2/I1	T1404
	399	0.00303	H1890/X1	K1383/N16/R3/E1/T1
	430^T^	0.00605	E1889/D1/K1	E1404
	473^T^	0.00803	N1890/D1	D1001/N403
	527^T^	0.00513	V1873/I12/A2/L2/X2	L1403/V1
	541^T^	0.00052	G1866/W12/H6/M3/Y1/-3	G1404
NA	2	0.00001	N1313/-79/I2/S1/X1	N1532/-200/K3
	6	0.00009	K1350/-42/N3/R3	K1634/R14/-87
	35	0.00151	S1392/G2/C1/I1/V1	S1733/N1/G1
	52^TG^	0.00510	S1397	R1686/K17/S17/N9/G4/X1/-1
	254	0.00188	K1396/X1	K1581/R154
	257	0.00119	R1388/K8/X1	K1735

^1^In this column, B indicates that the site lies in the B-cell antigenic regions [18]. G means that it is a potential glycosylation site. T indicates that the site lies in the T-cell antigenic regions [19]. R indicates that it is a receptor-binding site [11]. We use the same numbering strategy for HA as Deem and Pan [18] and start numbering from the amino acids DTLC. The NA numbering starts from MNPN.

^2^In these columns, capital letters stand for amino acids and numbers following them indicate the number of times they occur in the alignment. X indicates codons that are not translated properly.

Between North American swine strains and the pandemic 2009 human strains, 25 sites in HA were differentially selected, with 16 in HA1 and 9 in HA2 (Table 4). Among them, 19 sites lie in T-cell and/or B-cell antigenic regions. For example, positions 34, 48, 154, 189, 205, 207, 223, 263 and 306 in HA1 are located in the B-cell antigenic regions, while positions 31, 32, 34, 154 in HA1 and 411, 427, 434, 458, 478, 479, 530, 547 are located in the T-cell antigenic regions. In particular, position 223 is among the key sites able to affect receptor-binding ability [11]. Different amino acid polymorphism has also been seen at a few positions, such as 203, 205, 207 and 374 (Table 4).

**Table 4:** Sites in hemagglutinin under differential selection between isolates from North American swine and the pandemic 2009

**Position**^**1**^	P-value	**Amino acid polymorphism**^**2**^	
		
		North American swine	Pandemic 2009
31^T^	0.00512	N196	N1879/D8/X3/S1
32^T^	0.00222	L196	L1795/I94/X2
34^BT^	0.00190	E196	E1886/G2/X2/K1
39	0.00266	G196	G1887/E3/R1
48^B^	0.00008	A196	A1879/X6/T3/V1/P1/S1
154^BT^	0.00719	K195/R1	K1885/E2/X2/N1/T1
189^B^	0.00876	Q192/E3/L1	Q1891
197	0.00014	A192/T3/S1	A1851/T23/S17
203	0.00519	S178/T17/P1	T1341/S542/X7/A1
205^B^	0.00828	K166/R20/T8/Q2	R1852/K32/G2/X2/T1
207^B^	0.00286	N144/S43/Y5/D4	S1891
223^BR^	0.00199	Q196	Q1858/R18/X15
232	0.00890	T196	T1887/A1/I1/K1/X1
263^B^	0.00231	S196	S1889/F1/P1
304	0.00468	P196	P1875/S15/X1
306^B^	0.00929	Y196	Y1888/H3
374	0.00001	G183/R13	E1608/K275/G8
411^T^	0.00062	V196	V1839/I51/X1
427^T^	0.00159	V196	V1886/I5
434^T^	0.00550	T196	T1885/N4/X2
458^T^	0.00687	K196	K1885/N3/R2/E1
478^T^	0.00558	S196	S1889/G1/N1
479^T^	0.00355	V180/I16	V1891
530^T^	0.00409	L196	L1889/G1/X1
547^T^	0.00645	I195/-1	I1847/-22/K8/V5/T4/T3/C1/X1

^1^In this column, B indicates that the site lies in the B-cell antigenic regions [18]. T indicates that the site lies in the T-cell antigenic regions [19]. R indicates that it is a receptor-binding site [11]. We use the same numbering strategy as Deem and Pan [18] and start numbering from the amino acids DTLC.

^2^In these columns, capital letters stand for amino acids and numbers following them indicate the number of times they occur in the alignment. X indicates codons that are not translated properly.

In addition, between the Eurasian swine isolates and the pandemic 2009 isolates NA, there were five sites under distinctive selection, with three lying in the T-cell antigenic regions (Table 5). Among them, 321, 453 and 454 are within the T-cell antigenic regions [19]. Although differential selection between the two lineages has not led to distinct amino acid polymorphism, the pandemic 2009 strains did display a greater degree of amino acid polymorphism at positions 35, 381, 452 and 453 (Table 5).

**Table 5:** Sites in neuraminidase under differential selection between isolates from Eurasian swine and the pandemic 2009

**Position**^**1**^	P-value	**Amino acid polymorphism**^**2**^	
		
		Eurasian Swine	Pandemic 2009
35	0.00312	S92	S1392/G2/C1/I1/V1
321^T^	0.00425	I58/V34	I1395/X2
381	0.00634	T92	T1391/I4/N2
452^T^	0.00049	T92	T1385/-7/A2/I1/S1/N1
453^T^	0.00003	V92	V1383/-8/M4/G2

^1^In this column, T indicates that the site lies in the T-cell antigenic regions [19]. The NA numbering starts from MNPN.

^2^In these columns, capital letters stand for amino acids and numbers following them indicate the number of times they occur in the alignment. X indicates codons that are not translated properly.

## Discussion

In the present study, we investigated the positive selection pressures acting on HA and NA proteins of H1N1 influenza viruses. Despite the fact that the global ω for each cluster was below 1, a site-by-site analysis showed that some amino acid positions were under positive selection. Our results suggest that the pandemic 2009 human isolates have been subject to the strongest positive selection. Positive selection on HAs and NAs of isolates from humans was stronger than that on the swine strains. The avian strains were subject to the weakest selection, with no site found to be positively selected in avian isolates for either HA or NA. This indicates differing degrees of selection pressures acting on viruses from different hosts.

Although the HA2 domain also has important biological functions [5], a site-by-site positive-selection analysis of this domain has seldom been mentioned in previous studies [10,13]. We found some positively selected sites in the HA2 domain and this is consistent with a previous report [13]. Some of them are located in T-cell antigenic regions, such as 411, 451, 460 and 530 (Table 1). Therefore, positive selection on the HA2 domain might be responsible for the antigenic variation of the viruses. In particular, position 399, which was reported to be associated with host adaptation of the virus, has also been detected to be under positive selection [20]. However, for the amino acids in the HA2 subunit previously reported to be associated with host adaptation, we found no evidence of positive selection among the human H1N1 influenza viruses [20]. Therefore, based on current evidence, a major contribution of the HA2 domain to the survival of the pandemic 2009 strains might involve the antigenic variation resulting from positive selection.

Similar to the findings of Furuse et al. [13], our results reveal that the pandemic 2009 human strains were subject to different selection pressures compared to seasonal human strains. Twenty-four HA sites and six NA sites were differentially selected. Most of these sites lie in the B-cell and/or T-cell antigenic regions. However, both the SLAC and FEL methods showed that 222 and 451 were positively selected for human strains. Position 222 is situated within B-cell antigenic regions and is also associated with receptor binding. Position 451 is located within the T-cell antigenic regions of HA2. However, selection at these two positions was not detected in the previous studies [11,13]. This might be explained by the larger sample size in the present study. That many positively selected sites are located in the T-cell and/or B-cell antigenic regions might indicate that positive selection from the hosts, perhaps caused by vaccination and mass use of anti-viral drugs, might lead to corresponding variations in the T-cell and/or B-cell antigenic regions of the viruses. Accordingly, this would reduce the efficacy of vaccines and increase viral fitness.

Many amino acids have been reported to be associated with the host shifts of the viruses from birds to humans [20]. Although both the seasonal human H1N1 and the pandemic 2009 viruses did not come directly from avian hosts, some positively selected positions that have also been previously reported to facilitate the inter-host transmission of the virus showed distinct amino acid polymorphism (Table 3). Although most of the viruses of these two lineages had D187, the amino acid polymorphism was more diverse for the seasonal H1N1 lineage, with at least seven different amino acids appearing at this position. At position 399 in HA2, the seasonal strains showed greater amino acid variation, with 1383 sequences possessing K, whereas the majority of the pandemic strains had H. In particular, the avian viruses had E187 and N399, whereas viruses from pigs had D187 and H399. Therefore, the E to D mutation at position 187 and N to H mutation at position 399 might have facilitated the inter-transmission of the virus from birds to pigs and also helped the virus to adapt to humans.

Previous work has also shown that sites 138, 186, 190, 194, 225, 226 and 228 in HA1 are key positions concerning the receptor-binding property [11]. Our results revealed that 190 and 225 (numbering 187 and 222 in this study) were positively selected for seasonal human H1N1 and the pandemic 2009 H1N1, respectively. In addition, position 226 (numbering 223 in this study) was differentially selected between the pandemic 2009 H1N1 and the North American swine H1N1. Positive and/or differential selection has caused significant amino acid polymorphism at these positions and this might favour the inter-host transmission of the viruses from pigs to humans.

Differential-selection analysis also supported the pandemic 2009 strains being subject to distinctive selection compared to their progenitors. Specifically, different selection pressures have acted on HA proteins of the pandemic 2009 human and North American swine strains. Many of these differentially selected sites are located in the T-cell and/or B-cell antigenic regions. Similarly, selection pressures on NA proteins of the pandemic 2009 human viruses differed from those on the Eurasian swine strains and some positions are located in T-cell antigenic regions. Different selection pressures have caused amino acid variations at these positions. These might account for the antigenic variation of the pandemic 2009 human viruses with those from pigs.

The N-linked glycosylation is noteworthy because of its ability to influence virus survival and virulence [22]. Robertson et al. [23] suggest that mutation at site 160, resulting in the loss of a glycosylation site, could cause the antigenic drift. This site has also been considered to be the candidate amino acid for loss of the ability to agglutinate chicken erythrocytes [24]. Our results revealed that some glycosylation sites were under positive selection, such as positions 160 and 162 in HA, or differential selection, such as position 52 in NA. Considering that HA sites 160, 162 also lie in the B-cell antigenic region, positive selection at these two sites might play a greater role in viral adaptation. Site 52 in NA is also noteworthy. In the seasonal human strains, less than 10% of isolates had S52. However, all of the pandemic 2009 human strains possessed S52. Therefore, this potential glycosylation site might also contribute to the prevalence of the pandemic 2009 strains.

It has been reported that mutations at some NA sites are associated with drug resistance of the strains. For example, H274Y and N294S confer resistance to oseltamivir [7]. Janies et al. [14] reported that positive selection on position 274 was responsible for the wide spread of the drug-resistant strains of both seasonal and pandemic H1N1 lineages. Herein, we found evidence of positive selection acting on position 274 (numbering 275 in this study), suggesting that positive selection did play a significant role in the emergence and prevalence of the drug-resistant variants of seasonal human H1N1 lineage [14]. However, there was limited amino acid polymorphism at position 274 and more than 98% (n = 1372) of the pandemic H1N1 strains possessed H at this position. Neither the SLAC nor FEL analysis found position 274 to be under positive selection (Table 2). Therefore, positive selection might not be responsible for the spread of the oseltamivir-resistance of the pandemic strains.

Compared to the findings of Janies et al. [14], our results revealed a greater number of sites of NA proteins to be under positive selection. Both the SLAC and FEL analyses produced evidence of positive selection at positions 84, 151 and 382. In particular, mutation at position 382 has been reported to be involved in facilitating host shift of the virus. Together with the fact that some positively selected sites of NA proteins are situated in B-cell antigenic regions, and associated with drug resistance, it is possible that positive selection on NA proteins has had a profound effect on the seasonal human H1N1 viruses.

As shown in our analysis and in other previous reports, there is no distinct lineage displacement for the pandemic 2009 cluster in the HA and NA trees (Figures S1 and S2). This does not agree with the hypothesis that stronger positive selection usually leads to lineage displacement. This phenomenon may be explained by the low global ω value for the pandemic 2009 cluster (0.34 for HA and 0.27 for NA), although it is the highest among the values for all five clusters (Figures 1 and 2). This indicates that although some amino acid positions are subject to positive selection, most of the positions are evolving neutrally or are under negative selection.

## Conclusions

Our analysis shows that the HA2 domain and NA have been under positive selection. Although we only found indications of weak positive selection acting on the whole HA and NA proteins, the pandemic 2009 strains were subject to the strongest selection, differing from those on the seasonal human H1N1 viruses, North American swine viruses and Eurasian swine viruses. Most of the positively selected sites were located in the antigenic regions or were sites with known functional importance. This might account for the altered pathogenic profile of the pandemic 2009 strains and might have helped them to better adapt to the new hosts. In addition, our findings suggest that selection pressure on position 274 of NA protein, a site associated with drug resistance, might be responsible for the prevalence of the drug-resistant variants of the seasonal human H1N1 lineage.

## Methods

### Datasets

All HA and NA gene sequences of H1N1 influenza A virus for this analysis were retrieved from the NCBI Influenza Virus Resource (using H1 and N1 subtype as search queries) [25]. Two datasets were compiled: Dataset1) all HA genes from human, swine, and avian strains; Dataset2) all NA genes from human, swine, and avian strains. Redundant sequences were removed.

Each dataset was aligned under the open reading frame using the HyPhy 2.0 software package [26]. We then constructed a maximum-likelihood tree using RAxML for each dataset, assuming the GAMMACAT substitution model and setting the 1918 human sequence as the outgroup [27]. A rapid bootstrapping analysis was conducted using 1000 replicates, with other parameters set to the default values. Based on the resulting maximum-likelihood tree, we further divided Dataset1 and Dataset2 into ten subsets (Tables 1 and 2): 1.1) all HA genes from avian strains; 1.2) all HA genes from North American swine strains; 1.3) all HA genes from Eurasian swine strains; 1.4) all HA genes from seasonal human strains; 1.5) all HA genes from pandemic 2009 strains; 2.1) all NA genes from avian strains; 2.2) all NA genes from North American swine strains; 2.3) all NA genes from Eurasian swine strains; 2.4) all NA genes from seasonal human strains; 2.5) all NA genes from pandemic 2009 human strains.

### Analysis of positive selection

Global ω was calculated for each cluster using HyPhy. Maximum-likelihood trees obtained from the previous step were set as input trees. The MG94REV3x4 substitution model was applied, using equal equilibrium frequencies. The single likelihood ancestor counting (SLAC) method is appropriate for large alignments but might underestimate the number of positively selected sites, whereas the fixed-effects likelihood (FEL) method takes rate variation of synonymous and nonsynonymous rate into account and can be efficiently parallelized [28]. For the sake of comparison, we chose to use both of these methods [26,28]. Maximum-likelihood trees estimated by RAxML in the previous step were set as the input trees. The HKY85 model was selected as the best-fitting model of sequence evolution. A global ω value was estimated using a codon model obtained by combining the MG94 and HKY85 models. A two-rate FEL model was applied, allowing *d*_*N *_and *d*_*S *_to be adjusted across sites. P-values of <0.05 were considered to be significant.

### Analysis of differential selection

A comparative selection test was applied for datasets 1.4 and 1.5, and datasets 2.4 and 2.5, to detect codons under differential selection between seasonal human H1N1 and the pandemic 2009 strains. The same analysis was also done between the pandemic 2009 human strains and those from North American swine for HA, and between the pandemic 2009 human strains and those from Eurasian swine for NA. This was performed in HyPhy by using the standard analysis procedure - "CompareSelectivePressure.bf". All settings for the comparative selection analysis were the same as those in the previous selection analysis. P-values of <0.01 were considered to be significant.

## Competing interests

The authors declare that they have no competing interests.

## Authors' contributions

Conceived and designed the experiments: WS, CZ. Performed the experiments: WL, HQ. Analyzed the data: WL, WS. Wrote the paper: WL, WS. Revised the paper: SYWH, AL, YZ and CZ. All the authors read and approved the final manuscript.

## Supplementary Material

Additional file 1 **Phylogenetic tree estimated using HA genes from all hosts**. Colours indicate different clusters: Orange (1.1, avian strains); Blue (1.2, North American swine strains); Pink (1.3, Eurasian swine strains); Green (1.4, seasonal human strains); Red (1.5, the pandemic 2009 human strains). Numbers above the main nodes denote bootstrap values. Click here for file

Additional file 2 **Phylogenetic tree estimated using NA genes from all hosts**. Colours indicate different clusters: Orange (2.1, avian strains); Blue (cluster 2.2, North American swine strains); Pink (2.3, Eurasian swine strains); Green (2.4, seasonal human strains); Red (2.5, the pandemic 2009 human strains). Numbers above the main nodes denote bootstrap values. Click here for file
