# Supplementary figures and images for: Positive selection on hemagglutinin and neuraminidase genes of H1N1 influenza viruses
Source: Virol J. 2011 Apr 21;8:183. doi: 10.1186/1743-422X-8-183 (PMC3094300; doi:10.1186/1743-422X-8-183)

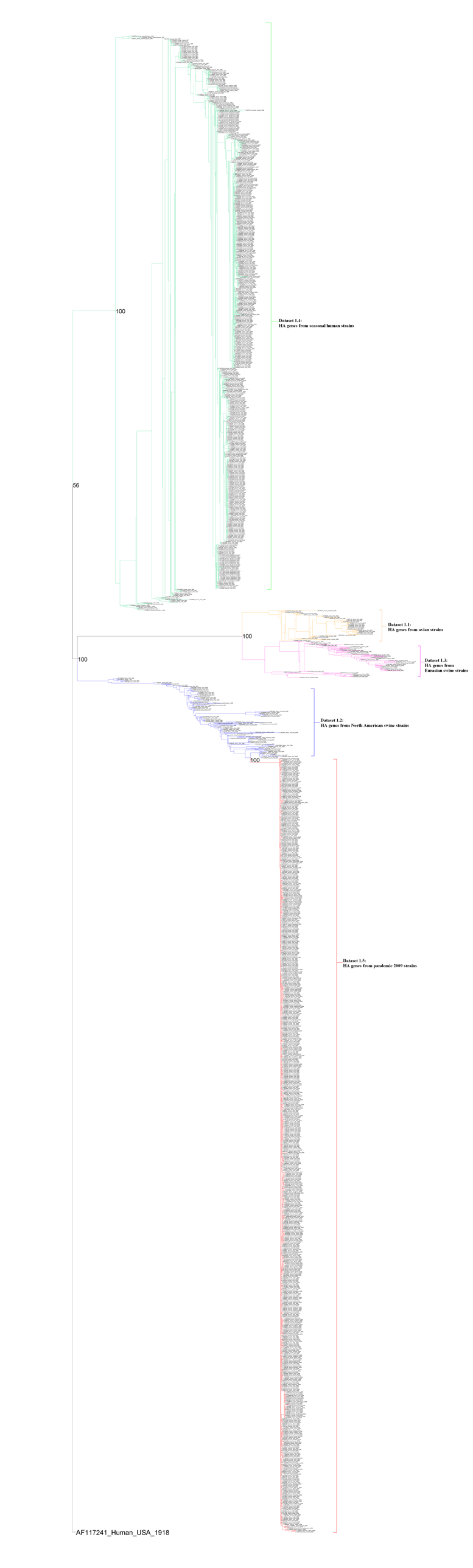

Supplement: Additional file 1 — Phylogenetic tree estimated using HA genes from all hosts. Colours indicate different clusters: Orange (1.1, avian strains); Blue (1.2, North American swine strains); Pink (1.3, Eurasian swine strains); Green (1.4, seasonal human strains); Red (1.5, the pandemic 2009 human strains). Numbers above the main nodes denote bootstrap values. [file 1743-422X-8-183-S1.TIFF]
